# Bisphosphonate-Induced Osteonecrosis of the Maxilla Resembling a Persistent Endodontic Lesion

**DOI:** 10.7508/iej.2016.01.013

**Published:** 2015-12-24

**Authors:** Hossein Mosaferi, Mahta Fazlyab, Sanaz Sharifi, Sepideh Rahimian

**Affiliations:** a *Oral and Maxillofacial Radiology Department, Shahid Beheshti University of Medical Sciences, Dental School, International Branch, Tehran, Iran; *; b * Department of Endodontics, Dental Branch, Islamic Azad University of Medical Sciences, Tehran, Iran;*; c * Iranian Center for Endodontic Research, Research Institute of Dental Sciences, Shahid Beheshti University of Medical Sciences, Tehran, Iran; *; d * Oral and Maxillofacial Radiology, Dental School, Jondy Shapoor University, Ahvaz, Iran*

**Keywords:** Bisphosphonate, Breast Cancer, Osteonecrosis, Periapical Pathosis

## Abstract

A 52-year-old Caucasian woman suffering from pain in the anterior maxillary region, presented to the clinic. Examination revealed a draining sinus tract in the buccal vestibule of the maxilla in the left anterior segment and expansion in the middle of palate. On conventional radiographic examination the lesion was initially assumed to be a periapical problem related to the incisors but subsequently it was diagnosed to be a bisphosphonate osteonecrosis.

**Conclusion:**

Acquiring a comprehensive medical history from the patients, conducting the clinical vitality tests and most importantly being familiar with the non-odontogenic lesions that can be side effects of specific medications are important requirements for reaching a correct diagnosis.

## Introduction

Many malignancies have tendency to involve the surrounding/distant bone structures as in 75% of the patients with breast cancer, bone metastases occur [1]. The metastatic lesions are sometimes accompanied by skeletal morbidity and severe pain. Prevention of these complications subsequent to cancer diagnosis is very important [2]. 

Medicines from bisphosphonate group are used for treatment of metabolic bone diseases (*e.g.* Paget disease), osteoporosis, multiple myeloma, *etc.* [3, 4] and are considered as the standard modality in prevention of skeletal complications in patients with bone metastases [5]. The mechanism of action is through inhibition of osteoclastic bone resorption and prevention of tumor cells proliferation and angiogenesis [3].

Intravenous zoledronic acid (zoledronate) was the first introduced bisphosphonate for treatment of bone metastases after the breast carcinoma [2] and was approved for prevention of skeletal lesions in patients with multiple myeloma or bone metastases of any solid tumor [5, 6]. However, during the past years, bisphosphonates, especially intravenous preparations, are held responsible for osteonecrosis of the jaws (ONJ) [3]. ONJ was first described in 2003 [7], as the transmucosal exposure of jaw bone for at least eight weeks, in a patient with history of bisphosphonate consumption without any history of radiotherapy in the head and neck region [8]. In most of the cases bone exposure is clinically obvious [9].

Based on retrospective studies, the rate of bisphosphonate-related ONJ is up to 19% in patients with bone metastasis [9] and zoledronate is more dominantly responsible for ONJ [2]. The mandible is more commonly affected than the maxilla with a 2:1 ratio [3, 9]. The present case describes the challenges brought to the clinicians during examination and diagnosis of a bone lesion surrounding the left maxillary canine that was diagnosed as bisphosphonate-related ONJ after many sessions of orthograde and retrograde endodontic treatments.

## Case Report

A 52-year old female with her chief complaint being pain in anterior left maxillary region, was referred to a private maxillofacial imaging center, by a general practitioner. 

**Figure 1:**
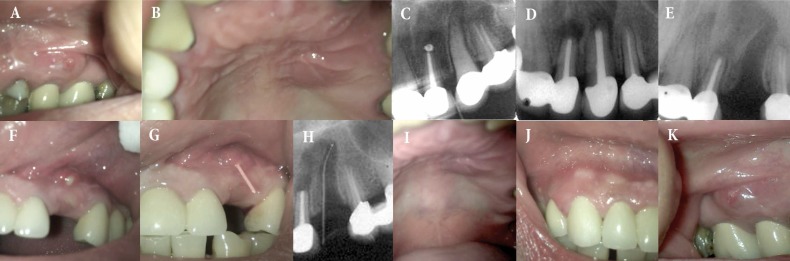
*A and B)* The presence of sinus tract and swelling in the left lateral incisor and canine in the buccal vestibule and palate; *C)* Periapical lucency surrounding the left lateral incisor and canine; *D) *Persistent lesions after orthograde treatment led to periapical surgery; *E) *Extraction of the canine; *F-I) *Reoccurrence of the sinus tract and palatal swelling; *J and K) *Appearance of similar lesions on the right maxillary side

According to the dentist, she had a history of breast cancer, mastectomy and chemotherapy. During clinical examination of the buccal vestibule in the left lateral and canine region, a draining sinus tract was detected. Also in the middle of palate a firm pediculate expansion was seen (Figures 1A and B). No other clinical sign was present. The canine, like other anterior teeth, was covered with full PFM restoration.

For more evaluation periapical radiography was ordered. The images revealed periapical radiolucency and widening of the periodontal ligament (PDL) surrounding the left lateral incisor and canine (Figure 1C). The teeth were not endodontically treated and considering the clinical and radiographic manifestations, two-session root canal therapy with two-week calcium hydroxide intracanal medication was performed. However, signs and symptoms did not resolve and periapical surgery was indicated (Figure 1D). 

After surgery, 500 mg amoxicillin and 250 mg metronidazole was prescribed every 8 h for seven days. During the couple of weeks after surgery the patient reported no complications. However, she returned shortly after the end of antibiotic therapy, due to recurrence of pain and pus drainage. Finally the dentist decided to extract the canine (Figure 1E). Unfortunately, after one week not only the sinus tract re-occurred on the left side of maxilla (Figures 1F to I) but also similar lesions appeared on the right maxillary posterior segment (Figures 1J and K).

**Figure 2:**
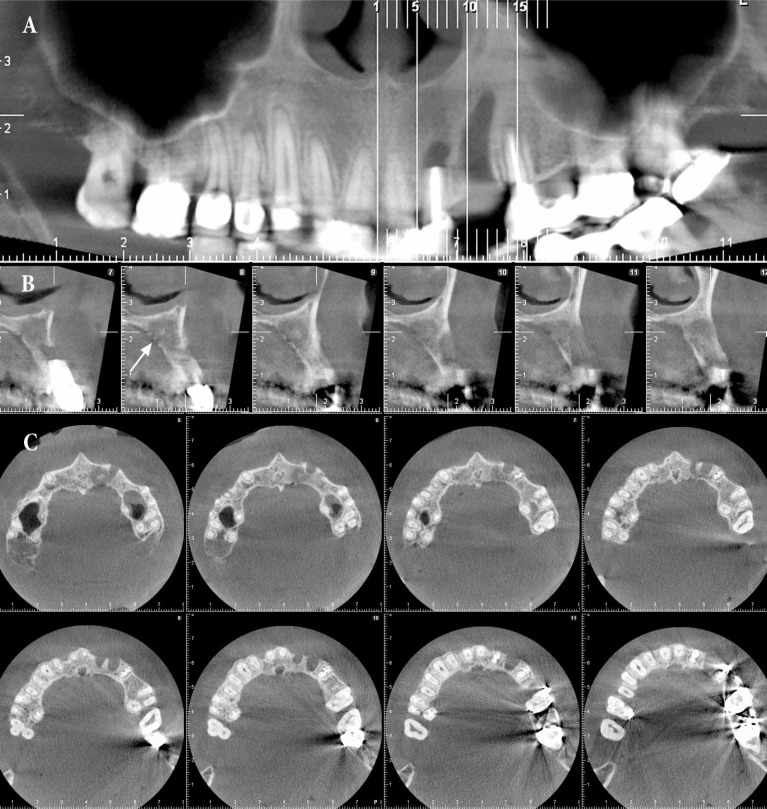
*A)* Generalized PDL widening in anterior maxilla and sclerosis of trabecular bone; *B *and* C)* Perforation of the palatal cortex in and sequester formation

The dentist referred the patient to the maxillofacial radiologist for more evaluation. After more assessment of the radiographies the generalized PDL widening in anterior maxilla and sclerosis of trabecular bone was detected (Figure 2A). Also; cone-beam computed tomography (CBCT) imaging was indicated (Figure 2B) which revealed the perforation of the palatal cortex in addition to the sequester formation (Figures 2B and C). Considering the clinical and para-clinical findings and patient’s medical history, she was questioned about consumption of additional non-declared medicine. She approved consumption of 4 mg/5 mL intra-venous zometa for the past four years to prevent bone metastasis. 

As a result the primary diagnosis was bisphosphonate-related ONJ and the patient was referred to an oral-maxillofacial surgeon in order to take a biopsy. Histological assessment showed areas of necrotic bone with signs of infection and also regions with increased bone turnover which was compatible with ONJ and confirmed the final diagnosis. The patient was referred for treatment.

## Discussion

The present report represented the challenging diagnosis of a bisphosphonate-related ONJ that had the clinical profile of a common endodontic lesion with draining sinus tract; after non-surgical and surgical endodontic intervention and persistence of the lesion the general practitioner suspected a non-odontogenic lesion. A revision through patient’s medical history (breast cancer and bisphosphonate therapy for prevention of bone metastasis) confirmed the final diagnosis.

The question is if there was a space for prompt diagnosis during the first visits by the dentist. The answer may be positive. The dentist failed to elicit the list of medicines consumed by the patient considering a history of malignancy, mastectomy and chemotherapy. But if the dentist was aware of patient’s injection of zometa, could he/she still consider the clinical relevance of it?

Bisphosphonates are analogous to synthetic pyrophosphate that concentrate in areas of bone and thus are powerful inhibitors of osteoclastic activity [3, 10] and have high affinity for hydroxyapatite crystals [3]. They have low intestinal absorption and are excreted through the kidneys without metabolic alteration [11]; as a result they are absorbed into the skeleton without degradation and have the incredibly long half-life of up to 12 years [3, 5]. Bisphosphonates cause resistance to osteoclastic activity [11] and are able to reduce the rate of pathological fractures, bone pain, the need for radiotherapy and surgery in patients with bone metastases [7, 9]. 

As the newer member of this family, amino-bisphosphonates (alendronate, risidronate, pamidronate, zoledronic acid and ibandronate) have several anti-tumor effects, such as induction of cell apoptosis, inhibition of cell adhesion to the extracellular matrix and inhibition of tumor invasion [12, 13].

The use of bisphosphonates in patients with multiple myeloma, primary and metastatic bone malignancies (breast, prostate, lung and renal cell carcinomas) [13], has reduced the skeletal complications, such as pathologic fractures, spinal cord compression, hypercalcemia secondary to malignancies and the need for bone radiotherapy or surgery [3, 14]. Our patient had a history of breast cancer and, although late, mentioned the consumption of intra-venous zoledronic acid for the past four years. In treatments exceeding 36 months with bisphosphonates the increasing risk of osteonecrosis increases which is more noticeable with the use of zoledronic acid and pamidronate [3].

Bisphosphonates have many oral complications: oral mucosal lesions arising from direct contact injury in cases of oral consumption [3] and induction of avascular osteonecrosis in maxilla and mandible [15]. In the present case, maxilla was involved unilaterally which became bilateral after some time. The only clinical manifestation was the presence of a sinus tract and a periradicular lucency around the crown-restored canine. ONJ has been reported as the exposure of mandibular (65%) or maxillary (26%) bone or both (9%) which is painless in one third of the cases [16] and has a tendency to occur on female patients and in posterior mandible [3]. In 40% of the cases it occurs spontaneously [7, 15] and bilateral involvement was slightly more common in the maxilla [3]. Clinically, intraoral lesions appear as areas of exposed yellowish hard bone with smooth or ragged borders and extra/intra oral sinus tracts may be present [9]. In our patient only sinus tract was present. In radiographies widening of the periodontal ligament is obvious which can be confusing and lead to misdiagnosis of periodontal issues [16]. We shall say if the dentist had radiographically evaluated the anterior segment, he/she would have detected the generalized PDL widening in our patient. Although this could be also attributed to necrotized crowned anterior teeth. The presence of full restorations challenged conducting pulp vitality tests. 

Poorly defined radiolucencies with moth-eaten borders, with or without radiopaque sequestra can be seen in advanced cases [3, 9], which was not detected in routine radiographies of the present case. Even surface colonization of the exposed bone by organisms of dental plaque such as *Actinomyces *can be detected by culturing, which is hard to distinguish from a true suppurative infection such as osteomyelitis [3]. Among the routine signs of ONJ including pain, swelling, fever, purulent secretion and oroanthral sinus tracts [15], our patient presented only the last two signs.

There are many medical conditions that can alter the procedure of routine dental treatments. The fact that if the patient’s problem was diagnosed earlier periapical surgery would not be chosen, emphasizes on the necessity of meticulous history taking before diagnosis and treatment.

## Conclusion

Although diagnosis of this case was particularly challenging, it was finally achieved. Taking a comprehensive history, especially from medically compromised patients and a complete list of patients’ medicines in addition to pulp vitality tests can help the clinician avoid “tunnel vision” during evaluation of the patient. This will provide the keys to successful diagnosis and treatment.
